# Experimental and Data Analysis Considerations for Three-Dimensional Mass Spectrometry Imaging in Biomedical Research

**DOI:** 10.1007/s11307-020-01541-5

**Published:** 2020-10-06

**Authors:** D. R. N. Vos, S. R. Ellis, B. Balluff, R. M. A. Heeren

**Affiliations:** 1 The Maastricht MultiModal Molecular Imaging Institute (M4I), Maastricht University, Universiteitssingel 50, 6229 ER Maastricht, The Netherlands; 2 Molecular Horizons and School of Chemistry and Molecular Bioscience, University of Wollongong, Wollongong, New South Wales 2522 Australia

**Keywords:** Mass spectrometry imaging, 3D imaging, Experimental set-up, Data analysis

## Abstract

Mass spectrometry imaging (MSI) enables the visualization of molecular distributions on complex surfaces. It has been extensively used in the field of biomedical research to investigate healthy and diseased tissues. Most of the MSI studies are conducted in a 2D fashion where only a single slice of the full sample volume is investigated. However, biological processes occur within a tissue volume and would ideally be investigated as a whole to gain a more comprehensive understanding of the spatial and molecular complexity of biological samples such as tissues and cells. Mass spectrometry imaging has therefore been expanded to the 3D realm whereby molecular distributions within a 3D sample can be visualized. The benefit of investigating volumetric data has led to a quick rise in the application of single-sample 3D-MSI investigations. Several experimental and data analysis aspects need to be considered to perform successful 3D-MSI studies. In this review, we discuss these aspects as well as ongoing developments that enable 3D-MSI to be routinely applied to multi-sample studies.

## Introduction

Mass spectrometry imaging (MSI) is a label-free molecular imaging technique for which no prior knowledge about the sample is needed. It enables the visualization of molecular distributions on solid surfaces using mass spectrometry by scanning the samples in a pixel-by-pixel manner where for each pixel a mass spectrum is generated. Visualization of molecular spatial distributions provides unique insights in many fields such as material science and biomedical research [1, 2]. In the latter, such surfaces are typically thin planar sections prepared from biological tissue. MSI has been used extensively to image the two-dimensional (2D) distributions of endogenous or exogenous (drugs and pharmaceuticals) compounds in such planar tissue sections for the study of tissue-based diseases, tissue pharmacokinetics, or the study of biomaterials in medical applications [3, 4]. However, the natural state of the original sample is volumetric, hence keeping the original three-dimensional (3D) information is important to be able to study the structural context of the sample in all dimensions.

In material science, where MSI has been employed since the 1960s [5], it is proven that 3D-MSI can provide essential information. Secondary ion mass spectrometry (SIMS), for instance, has been used extensively for 3D elemental analysis of semiconductors, superconductors, glass, stainless steel, solid oxide fuel cell components, aerospace alloys, coatings, and biomaterials [6, 7]. The unique capability of obtaining molecular depth profiles has provided evidence on how 3D-SIMS can aid in understanding complex volumetric structures [8–13].

The benefit of acquiring more relevant information through 3D-MSI is also of added value for biomedical research in which the added dimension can yield important contextual information about the biological tissue (see ‘Benefits of 3D-MSI’). In conventional 2D imaging, a chemical snapshot of a single tissue section is generated. This snapshot corresponds to a specific location in a larger sample. Tissue stereology [14] has shown that a single section may not be representative of the whole sample. Tissues are seldom homogeneous, and the mix of cells across several 2D planes varies strongly, especially in a diseased state [15]. This introduces a potential sampling bias. In clinical diagnostics, the discrepancy between the sampled sections and the variation across a whole tissue can lead to inaccurate predictions. This occurs especially in oncology where cancer can be spatially very heterogeneous [16]. This is corroborated by the use of radiomics in oncology, which has shown that 3D structural features contained in clinical *in vivo* scans harbor relevant clinical information [17]. An imaging feature was found for ovarian carcinoma that was predictive of the outcome after first chemotherapy. In glioblastoma, a specific imaging pattern was able to predict epidermal growth factor receptor (EGFR) overexpression. Another prime example for the necessity of volumetric investigation is the healthy brain which has therefore been the blueprint for many 3D omics studies [18].

Consequentially, 3D-MSI has already analyzed a variety of solid tumor tissues, rodent brains, and other organs, and applications are rapidly increasing [19, 20]. While these studies provide successful use cases, they also reveal technical aspects that require special attention and further development beyond conventional 2D-MSI. These aspects affect the whole study workflow and range from study design through sample preparation to data analysis. We will discuss them in this review, especially in the light of extending 3D-MSI to the analysis of larger sample (patient) cohorts to capture inter-individual effects. First, the most commonly used 3D-MSI techniques and their applications are briefly reviewed to illustrate the benefit of 3D-MSI in biomedical research. Then we highlight practical considerations in performing 3D experiments and 3D data analysis, as well as discuss remaining challenges.

## 3D Approaches in MSI

**Fig. 1.**
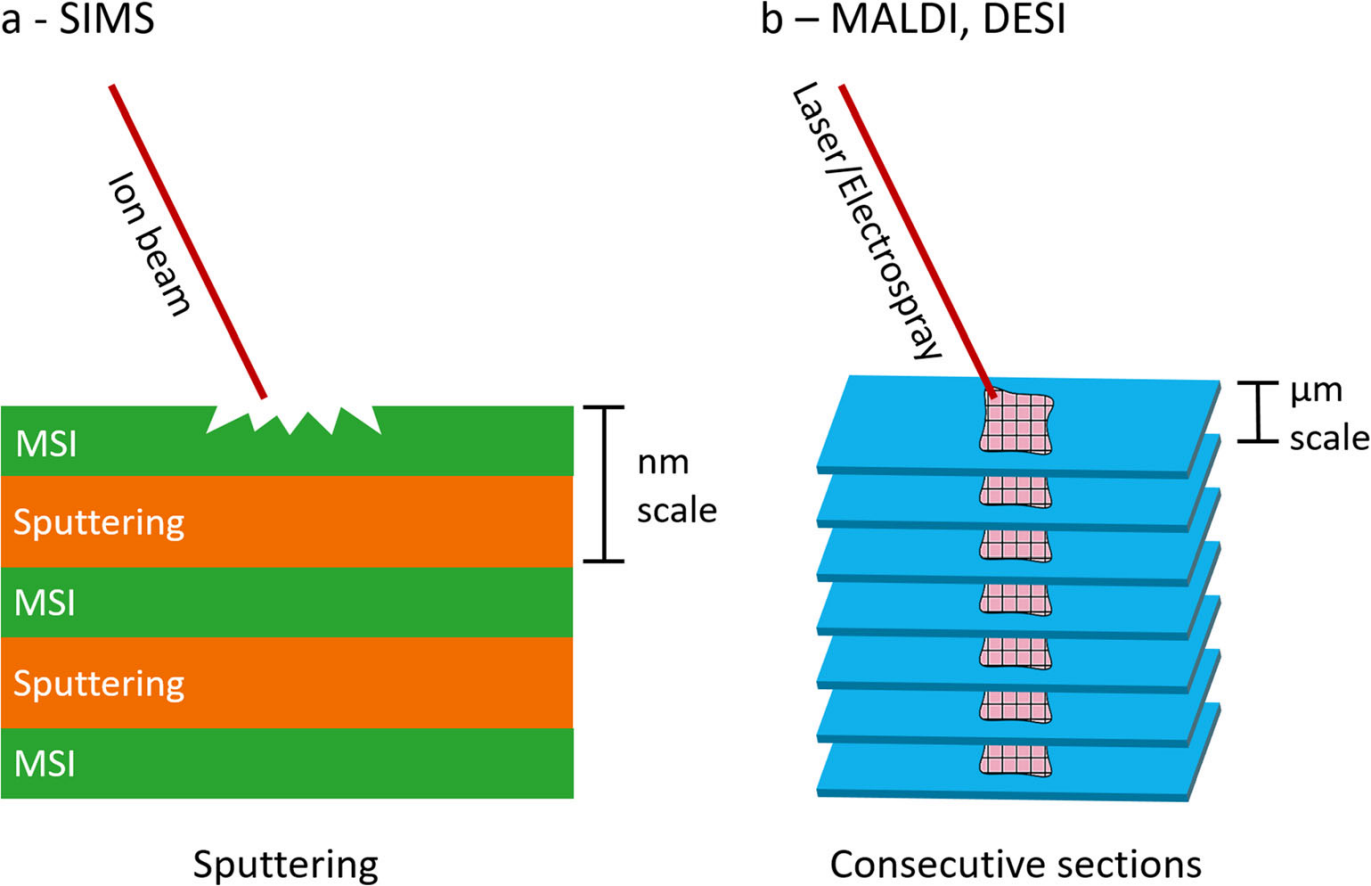
Schematic representation of the different approaches in 3D mass spectrometry imaging (MSI). **a** Secondary ion mass spectrometry (SIMS) continuously alternates imaging and sputtering cycles. **b** Matrix-assisted laser desorption/ionization (MALDI) or desorption electrospray ionization (DESI) rely on the sectioning of the sample into a stack of consecutive sections which are then analyzed individually and their data reassembled afterward.

There are generally two different ways by which 3D-MSI can be achieved, depending on the desorption/ionization technique used: via surface sputtering or via the analysis of multiple serial sections (Fig. 1).

In 3D-SIMS, surface imaging and surface sputtering, where the impacting primary ion beam removes a nanometer-thin layer during the sputtering cycle, are continuously alternated until the desired depth is reached (Fig. 1a). The depth-resolution depends on the type of ion beam and the associated sputter rate of the region being analyzed [21–23] but usually ranges between 10 [24] and 1 nm [25]. The use of polyatomic or cluster-based ion beams enables small-sized biomolecules, such as lipids, to be released intact from the surface. Additionally, the chemical, subsurface damage done by these beams is reduced or completely removed [2, 26]. This also allows an increased primary ion energy to be applied. A higher primary ion energy typically leads to higher secondary ion signal within a single pixel and results in improved image contrast [26]. The introduction of polyatomic ion guns, like the C_60_^+^ and Bi_3_^+^ ion guns with a smaller beam focus, even allows 3D analysis of single cells as it can be focused down to subcellular resolution and generates higher yields of intact biomolecules [26, 27].

Soft-ionization techniques, in contrast, do not rely on energetic sputtering but rather the soft desorption/extraction of biomolecules. In matrix-assisted laser desorption/ionization (MALDI) and desorption electrospray ionization (DESI), the two most common soft-ionization techniques used for MSI, a 3D image is obtained by serial measurements of consecutive sections from a sample (Fig. 1b). The final 3D dataset is then obtained via a reconstruction procedure. The overall sample volume that can be analyzed with these techniques in a given amount of time, is much higher than with SIMS. The analysis volume depends on the sectioning thickness (usually 10–20 μm) [28–30], the number of sections, and the spacing between the sections. MALDI and DESI are therefore often employed to measure whole tissues in 3D as a result of the large volume of analytical capabilities. However, compared with SIMS, the depth resolution between sections is limited by the thickness of the tissue section, which typically ranges from 4 μm (paraffin-embedded tissue) to 12 μm (frozen tissue). Molecules within a section are samples from an unknown extraction depth determined by the MALDI matrix or DESI solvent often assumed to be constant across a sample.

In MALDI-MSI, 3D applications have seen a recent rise in popularity due to advances in sample preparation and instrumentation, such as lasers with high repetition rates and fast-moving stages, which made high-throughput analysis possible [31–33]. As a consequence, whole tissue sections can now be measured within minutes, which allows a full 3D dataset to be acquired within a day.

Compared with MALDI-MSI, DESI-MSI requires less sample preparation as no external matrix is required to extract the molecules of interest. This does not necessarily mean a higher throughput for DESI-MSI over MALDI-MSI. Due to a limited spray focus, DESI has a lower spatial resolution than MALDI or SIMS. Various efforts are ongoing to increase the lateral spatial resolution that would allow for the acquisition of better quality images from different histological regions within a tissue [34].

## Benefits of 3D-MSI

Early 3D-MSI experiments have demonstrated feasibility and have paved the way for subsequent applications with different ionization modalities [35–37]. Here, we highlight selected applications of 3D-MSI that demonstrate its benefit or the need to contextualize the molecular information into an additional spatial dimension in certain situations.

### 3D SIMS-MSI

SIMS has been extensively used for 3D MSI studies of single cells and tissues as reviewed by Fletcher [11, 38]. Here, we highlight two applications focused on interfaces between drugs and surfaces. Biomaterials, like coronary stents, are frequently studied with three-dimensional SIMS. Coronary stents are coated with a polymer that contains an anti-inflammatory drug that is released over time to prevent the blocking of the stent. To visualize the distribution of the drug sirolimus in a stent, drug/matrix-coated metal coupons were imaged with 3D-SIMS [39]. A gold ion beam was used for imaging in conjunction with a carbon-cluster (C60) ion beam for sputtering, which results in low residual molecular damage after sputtering. This is needed since sirolimus is a mid-sized (914 g/mol) pharmaceutical compound. Sputter rates have been determined under the same measurement conditions on a similar polymer material to allow accurate determination of the depth scale. It was found that large areas of the surface and subsurface channels were composed primarily of sirolimus, followed by a drug-depleted region, and lastly, a relative homogeneous drug distribution in the polymer matrix. Comparing these 3D distributions with the elution rates showed that elution occurs relatively quickly from the drug-enriched surface area while it proceeds more gradually for the subsurface regions.

**Fig. 2.**
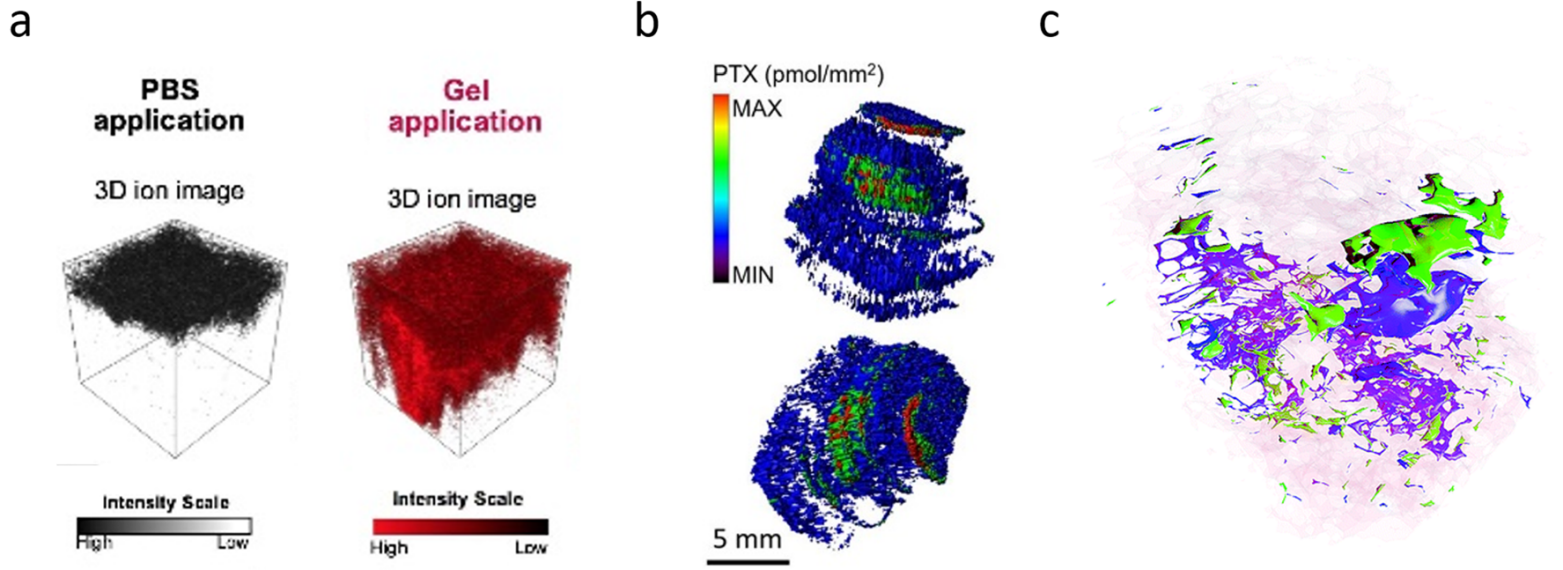
Applications of 3D mass spectrometry imaging (MSI). **a** 3D SIMS-MSI was used to study the permeation of ascorbic acid through *ex vivo* skin samples after the application of PBS or a hydrogel. The results showed a deeper permeation when a hydrogel is used. This article was published in [40] (copyright Elsevier). **b** Quantitative 3D MALDI-MSI of the anti-cancer drug paclitaxel in a malignant pleural mesothelioma tumor. Images indicate the drug is mostly located at the edge of the tumor. Figure adapted from [41] (licensed under CC BY 4.0: http://creativecommons.org/licenses/by/4.0/). **c** The application of advanced data analysis methods to a 3D DESI-MSI dataset of a human colorectal cancer sample revealed several metabolically different tumor subgroups highlighting the heterogeneity of tumors in three dimensions. Figure adapted from [19] (published by The Royal Society of Chemistry).

Supramolecular hydrogels and their capability to enhance skin permeation of vitamin C (ascorbic acid) and its precursor (ascorbyl glucoside) have also been investigated with 3D-SIMS of *ex vivo* porcine skin [40]. Researchers demonstrated that the hydrogel enhances skin permeation of both compounds and preserves the conversion of the precursor into vitamin c until it reaches the epidermal layer, the intended target (Fig. 2a). As the depth to the epidermis is *a priori* unknown and sputtering rates might change for each layers within a sample, the authors used two methods to calculate the *z*-resolution during sputtering. Optical profilometry was applied to determine the depth of the sputter craters. This provides an estimation of the average depth, and a phospholipid ion marker was monitored during sputtering to determine when the epidermis layer is reached.

### 3D MALDI-MSI

The application of 3D MALDI-MSI has seen a steady increase over the past years. Some of these have been discussed in earlier reviews, though this is only a small fraction [2, 42], thereby missing interesting papers like the investigation of pathogenesis in *Francisella* infection by Scott *et al.* [43] and the feasibility assessment of 3D MALDI-MSI with FTICR by Jones et al. [44]. One of the most investigated organs in the field of MALDI-MSI is the murine brain due to its molecular but morphologically well-charted complexity [45, 46]. The high-lipid content in the brain, which is a molecular class well compatible with MALDI- or DESI-MSI also contributes to the frequent usage of brain sections in MALDI-MSI.

Lipid changes upon traumatic brain injury were investigated in rat brain with 3D MALDI-MSI to gain more understanding of the biochemical alterations caused by this neurological damage that is a major cause of death and disability in children and young adults [47]. The researchers obtained rat brain tissue sections every 200–250 μm covering the entire area of the cortical-impact injury. Tissue sections were analyzed individually using MALDI-MSI of lipids at 70 μm spatial resolution, resulting in a voxel size of 70 × 70 × 220 μm in the final 3D dataset. The authors observed that certain lipids are specific for either the lesion site (*e.g.*, PC (42:9) (*m*/*z* 856.598)) or the ventricles (*e.g.*, PC (*m*/*z* 797.580)) that change from rostral to caudal while others co-localize with both regions (*e.g.*, *m*/*z* 741.545). Acquisition of the 3D model helped the authors gain more insight into the changes happening throughout the brain since the injury-related molecules are transferred via these ventricles throughout the brain and other organs. The 3D results provided a more global view of the impact of the traumatic brain injury and provided insight into pathological phenomena remote from the injury site.

With recent advances in instrumentation, especially in laser repetition rates, synchronized stage movement, and electronics, 3D-MSI datasets can now be generated much faster, up to 20–50 times, allowing the acquisition of biological or technical replicates. This was recently demonstrated by Paine *et al.*, who studied medulloblastoma, the most common malignant pediatric brain tumor, in six mouse brains by 3D MALDI-MSI of lipids. The voxel size was 50 × 50 × 150 μm, and a total of 223 sections were measured in this study [48]. A semi-supervised segmentation of all tissue sections was performed first to find the boundaries of the primary tumors to facilitate the analysis of this large amount of data. A comparison of metastasizing *vs.* nonmetastasizing primary tumors revealed ten lipids associated with medulloblastoma metastasis. As these lipids were observed to be very heterogeneous in their distribution throughout the tumor, single-section 2D-MSI could have missed the significance of these lipids due to sampling bias. This experiment demonstrated the added value of 3D-based analyses for tumor marker discovery and exploration. Even if multiple sections are investigated from one sample, it is important to prepare and analyze the sections in a reproducible manner to reduce inter-section technical variation. Paine *et al*. have ensured this by batch preparation (20–25 sections) and a quality assurance approach that involved tuning the signal intensity of each imaging run on the matrix to ensure similar ion yields on all sections [48].

This is especially true if MSI signals are to be compared quantitatively. For instance, Giordano *et al.* have employed 3D MALDI-MSI to quantify the distribution of the anti-cancer drug paclitaxel in mesothelioma tumor-bearing mice [41]. Tumor sections were taken every 500 μm, and MALDI-MSI experiments were performed at 100 μm lateral pixel size. The signal of paclitaxel was normalized to an isotopically labeled version of paclitaxel as an internal standard and quantified using a concentration series applied to a separate section. It was discovered that the distribution of the drug is influenced by the cellular heterogeneity of the tumor microenvironment showing its highest concentration at the edge of the tumor and a lower abundance in the center of the tumor with necrotic and fibrotic regions (Fig. 2b). The more accurate description of the distribution of the compounds obtained, demonstrates the added value of 3D-MSI for the quantitation of pharmaceutical compounds.

### 3D DESI-MSI

Few studies have so far used DESI in a 3D fashion. An early 3D DESI-MSI study has mapped the distribution of anabolic steroid esters through bovine muscle tissue previously injected with these compounds [49]. The spatial resolution in *x* and *y* was 500 μm, and the samples were spatially separated in the *z*-direction by 1 cm. The 3D profile was made at the injection site and demonstrates that the esters are indeed inside the tissue. This excludes external contamination which is an important element in the investigation of the illegal treatment of animals.

In a more recent study, the unique capability of MSI to describe intra-tumoral molecular heterogeneity has been for the first time extended to 3D by investigating a human colorectal adenocarcinoma biopsy by 3D DESI-MSI at 100 × 100 × 100 μm voxel size [19]. The application of advanced data analysis methods including deep learning and parametric t-SNE mapping helped to identify tumor subgroups and characteristic metabolites that were not detected by classical methods such as principal component analysis (Fig. 2c). This also exemplifies that, as the field of 3D-MSI is evolving, novel and advanced data analysis procedures are needed to extract all biological information from 3D-MSI data.

## 3D-MSI Experimental Considerations

The benefit of performing 3D mass spectrometry imaging also comes with some additional requirements and considerations in terms of sample preparation, experimental design, and data acquisition. The evaluation of these workflow elements is crucial to reliably correlate and compare findings throughout a 3D-MSI volume. Below, we will discuss several considerations that have a direct impact on the outcome of a 3D study.

### Single-Cell Preparation for SIMS

In the case of SIMS for single-cell analysis, cells need to be prepared in such a way that the 3D shape and integrity of the cells are maintained. One way of achieving this is by chemical fixation of the cells to preserve the cellular architecture of the cell [50–52]. In this process of fixation, certain molecules, like salts, can diffuse while proteins are cross-linked together [50, 52]. Another approach that retains the integrity of the cells and prevents diffusion of molecules is to prepare frozen-hydrated cells through cryo-fixation of cells in their native hydrated state [53]. This method achieves higher ion yields for certain chemical species but requires the instrumentation to be equipped with a cold stage to maintain the frozen-hydrated state of the sample throughout the entire workflow [53, 54]. UHV systems can result in the sublimation of water from the 3D samples which can subsequently deteriorate during cryo-imaging experiments. Alternatively, cells can be prepared for 3D-SIMS analyses by freeze fracturing where cells are trapped in a frozen sandwich, which is broken before analysis. This method maintains both cellular and molecular integrity but can lead to the loss of the top part of the cells and the fracture plane is not always reproducible. This makes it difficult to characterize both complementary fractured surfaces [53, 54]. All these sample preparation methods have their advantages (*e.g.*, maintaining cellular integrity, preventing diffusion) and drawbacks (*e.g.*, diffusion of molecules, unreproducible fracture planes), and choosing the best-suited method depends on the research question at hand.

### Serial Section Preparation

In the case of 3D-MALDI and 3D DESI-MSI, experiments might require the collection of dozens to hundreds of (semi-)consecutive sections from one sample. As a result, caution is required during sectioning to exclude the loss of individual sections and retain the appropriate order and spacing in the 3D stack. Keeping the samples in the same orientation in a consistent shape will, later on, simplify the alignment of the sections into a 3D volume. Minor misalignment of sections can be digitally corrected afterward [55]. This registration is often based on prominent morphological features, and heavy distortion can render molecular images unsuitable for inclusion in the 3D volume. This can be overcome by embedding the tissue in embedding materials like optimal cutting temperature (OCT) or gelatin [30, 56–58]. Care has to be taken that the right embedding material is chosen, compatible with the measurement. Embedding with OCT is generally not recommended for MSI as it contains a polymer which easily ionizes in positive-ion mode and may cause ion suppression or mask tissue-specific signals. Synthetic polymer-based embedding media also run the risk of smearing across tissue samples during sectioning [57]. Gelatin and CMC are biopolymer based and generate less spectral interference, which renders them more compatible with MSI experiments of biological surfaces [59]. Care has to be taken that biomolecules do not delocalize during the embedding process. Using an embedding medium also allows the use of fiducial markers that can aid in the registration process. Fiducial markers are placed in the embedding block and may help to determine the position, orientation, and distortion of each section. They are also very suitable for use in the co-registration of MSI data with images from different imaging modalities [60–62].

### Experiment Design

**Fig. 3.**
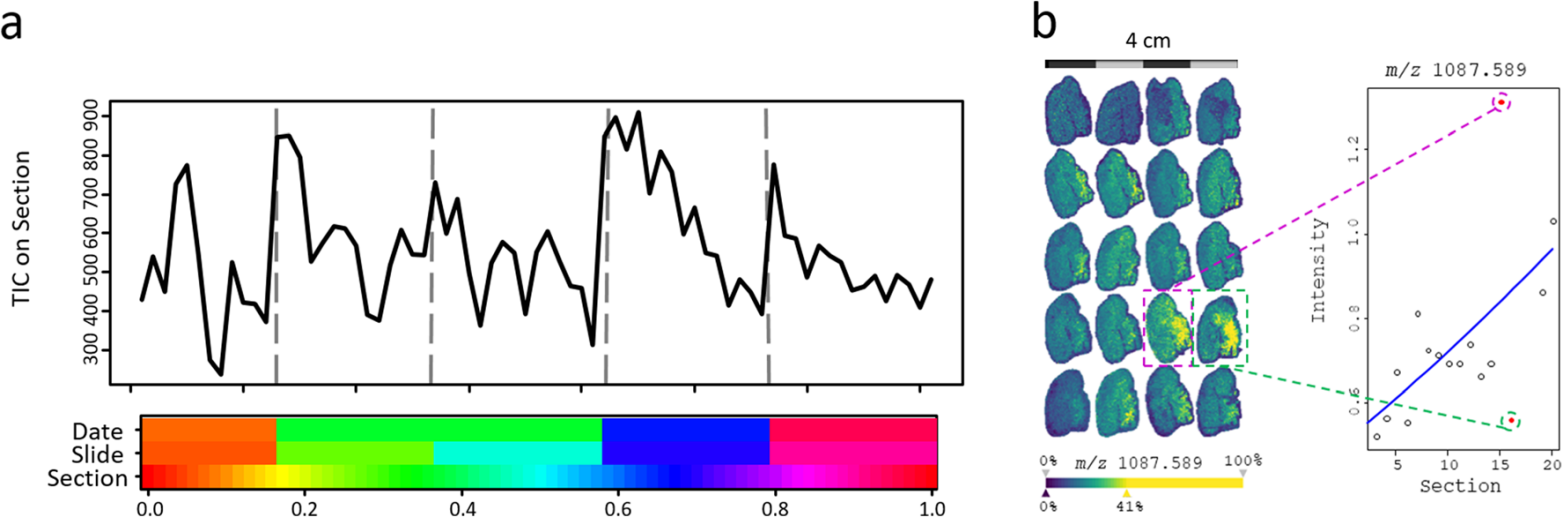
Experimental considerations in 3D-MSI. **a** When no randomization has been performed, the fluctuation of the overall signal intensity across every single section, represented by its total ion count (TIC), can be caused by other factors such as date of experiment, the slide, or the position of the section on the slide. **b** Assuming proper randomization and that close by sections should have similar molecular signals, we have recently proposed a z-direction based regression of molecular signals. Outliers are detected whose molecular signals are not in line with previous and successive sections. This article was published in [63] (copyright Elsevier).

3D-MALDI and 3D DESI-MSI experiments consist of the sequential measurement of serial tissue sections. If the sections or experiments have not been properly randomized, the entire contiguous parts of the 3D volume might be affected by a technical bias, which cannot be distinguished from a biological effect (Fig. 3a). It is, therefore, necessary to use randomization in both sample preparation and data acquisition. Serial sections should be distributed in a randomized fashion within and between the slides already during the sectioning process. This overcomes potential batch effects resulting from technical variance in slide preparation (cutting, on tissue chemistry, matrix application) and instrument-related batch effects such as a decrease or drift of system sensitivity during long runs of acquisition.

While randomization itself will certainly reduce a systematic technical bias that can be confused with a biological effect, it will not recognize technical outliers. This becomes even more important when *multiple* 3D datasets from different tissues are to be measured and compared. The total project execution duration exceeds classical 2D MSI projects by a factor corresponding to the average number of sections per 3D sample. The total sample preparation of a multi-sample 3D-MSI project can spread over weeks and months as only a single sample can usually be cut at a time. This increases the possibility of observing time-related batch effects between and within 3D-MSI datasets. A way to monitor experimental quality is the application and integration of quality control measurements. Quality control compounds can be applied before measurement or matrix application, thereby independently assessing either instrument performance or sample preparation, respectively. For instance, protein and peptide standards can be spotted onto the slides next to the tissue to determine matrix quality or digestion efficiency on a slide-by-slide basis [63, 64]. Unfortunately, this is not yet routinely applied in the field of 3D-MSI. Only recently, we have demonstrated the usefulness of quality controls to track digestion efficiency in a multipatient 3D-MSI study [63]. In this study, we incorporated cytochrome *c* as a quality control for digestion efficiency on a slide basis. By performing principal component analysis (PCA) on the cytrochome *c* spots and using a 95 % error ellipse, we could identify 11 slides on which the digestion was insufficient. This translated to 22 sections out of a total 280 measured giving a dropout rate of < 8 %. In addition to tracking sample preparation quality, it is also a necessity to monitor day-to-day instrumentation performance as instrument sensitivity can vary from one experiment to another. Monitoring instrumentation performance with a quality control compound measured over time can assist in the identification of technical outliers [65].

### Data Acquisition

Large volume measurements require careful planning of the total workflow, from sample preparation to data acquisition. Time can be a critical factor and influence the resulting 3D molecular dataset. Sample preparation should happen “just-in-time” before the measurements as otherwise, degradation of the samples might occur which might lead to false discoveries along the volume [66, 67]. Total acquisition time should be optimized to minimize the experimental variance within one 3D-MSI dataset while keeping throughput as high as possible. Dataset size is another critical factor. It increases linearly with the number of sections and samples and is quadratic ally dependent on the spatial resolution. The resulting total dataset size often determines the choice of optimal spatial resolution, the mass range, and the number of sections per sample. This also ensures during data analysis the various software packages and algorithms can read a full dataset in memory if so desired. We have recently proposed a procedure to determine the minimum number of sections needed to retain a minimum amount of the full information extracted from a 3D MALDI-MSI experiment [63]. This representativity analysis is based on calculating the correlation coefficient of a subset of samples to the full 3D dataset. In our study, we found that every third of the measured 20 sections were needed to reach a minimum correlation of 0.99 to the full 3D dataset. This way, a preselected, representative sample is defined to prospectively save sections from alike samples and reduce the data size of the study.

## 3D-MSI Data Analysis Considerations

### Outlier Detection

One of the challenges that MSI faces is the lack of a generic and reliable experimental outlier detection method to ensure a high degree of comparability for both inter- and intra-sample comparisons. Recently, we proposed a detection method to check for any possible outliers in 3D-MSI datasets. The method is based on *z*-directed regression analyses within a 3D volume and identifies sections as outliers whose molecular signals deviate statistically too much from the expected signal intensity (Fig. 3b) [63]. This method is generic and captures most experimental biases unless too many sections are affected at the same time from that bias thereby driving the regression analysis. This could be the case if many consecutive sections are on the same slide. These sections undergo all experimental steps at the same time and therefore all get affected by matrix application or a poor MS instrument performance at the same time.

### 3D Image Reconstruction

**Fig. 4.**
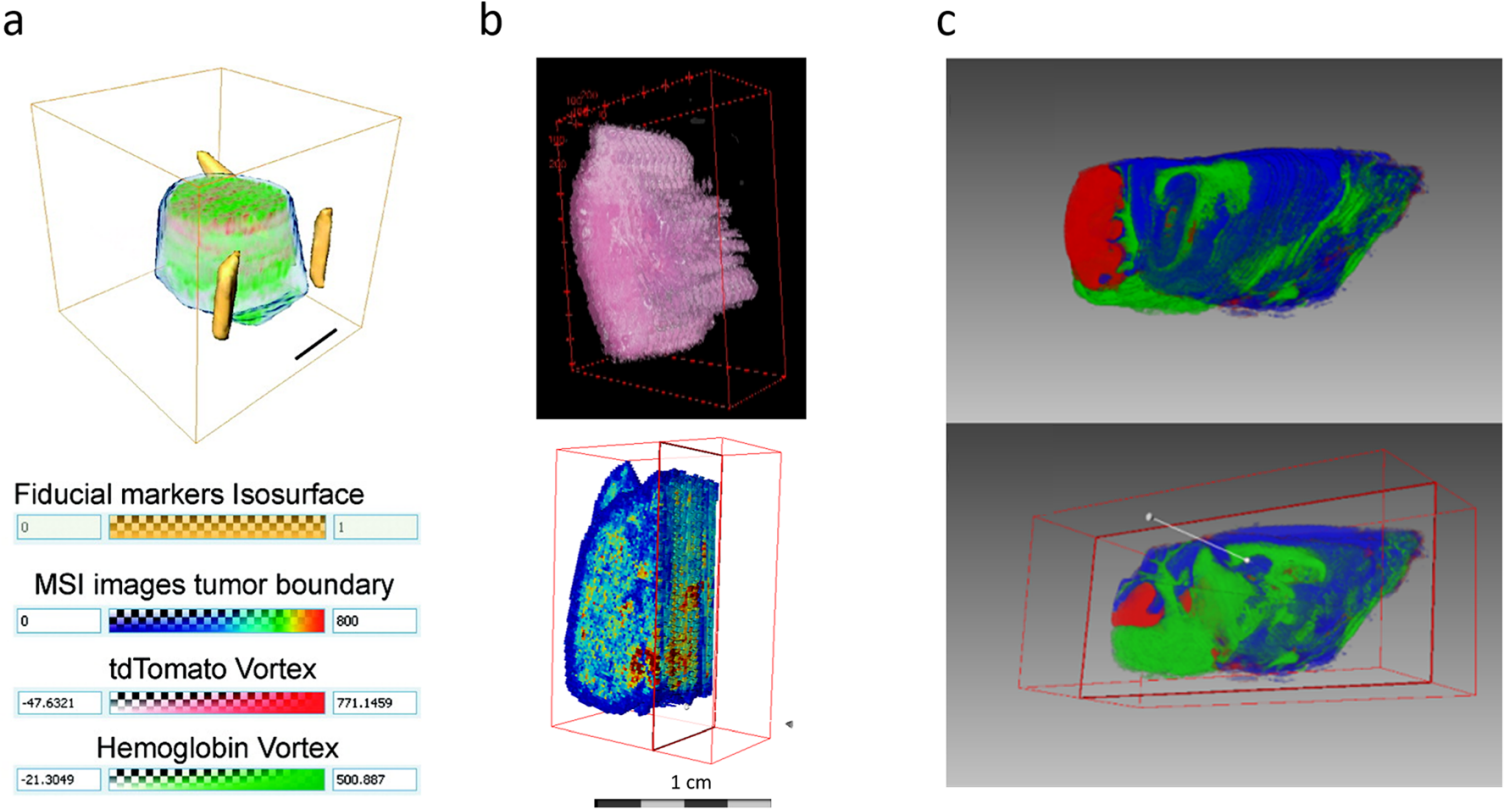
Alignment strategies in tissue-section-based 3D-MSI. **a** Alignment based on embedded fiducial markers requires a previous embedding of the sample into a medium but delivers good results. Adapted with permission from [60] (copyright American Chemical Society). **b** An alignment based on optical images requires the optical images to be linked to the MSI data before data analysis, *e.g.*, during the experiment. **c** Alignment based on only the MS images requires visible and well-defined structures in the images. The coarseness of these structures should match the spatial resolution of the MS images. Figure adapted from [48] (licensed under CC BY 4.0: http://creativecommons.org/licenses/by/4.0/).

**Fig. 5.**
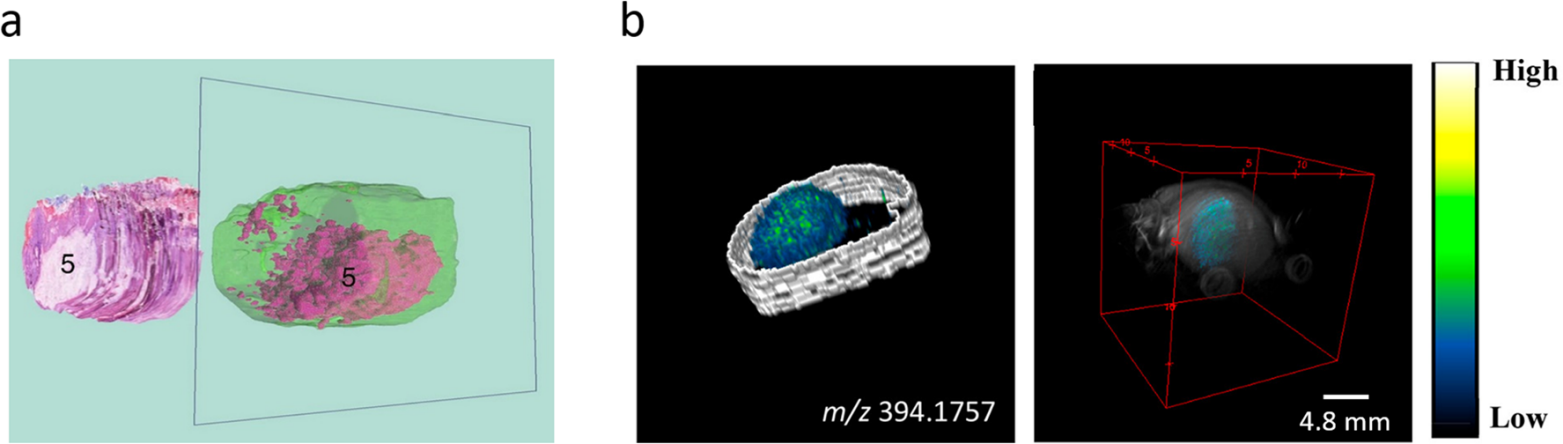
Integration of 3D-MSI with other modalities. **a** A 3D MALDI-MSI dataset consisting of 162 consecutive sections of an oral squamous cell carcinoma was combined with the corresponding 3D stack of histological images. Both volumes were placed in the same space allowing the co-visualization of both modalities at the same depth. This article was published in [70] (copyright Elsevier). **b** A 3D MALDI-MSI dataset of glioblastoma in a mouse brain was also combined with MRI. The visualization on the left shows a molecule that highlights the tumor area and on the right, the same ion is overlaid with the MRI image. Adapted with permission from [71] (copyright American Chemical Society).

It is of interest in many studies to align and stack the individual MSI images of a completed 3D-MSI dataset to construct a 3D volume, which can be used for an advanced interpretation of the spatial context of the molecular images. Embedded fiducial markers can be used as reference points to automatically co-register and stack the single sections (Fig. 4a) [60]. However, the 3D volume reconstruction can be completed without fiducial markers. This is often performed manually by using either anatomical features in optical images that are already aligned to the MS images [68, 69] or well-structured MS images to spatially align the consecutive MSI slices (Fig. 5b, c) [47, 48]. These approaches are only suitable for tissues with well-defined and visible structures in the optical or MS images. For the task of aligning and reconstructing 3D visualizations, different software applications have been used [37, 72–74], including ImageJ (https://imagej.nih.gov/ij/) [75], R software (https://www.r-project.org/), SCiLS Lab (Bruker Daltonik GmbH, Bremen, Germany), or Autoaligner® combined with Imaris (both from Bitplane, Zurich, Switzerland) [41]. With Autoaligner® image features are used to align the sections which are built into a volume with Imaris.[73]. In the case of ImageJ, there are different ways to align and can be done by first aligning stained sections using an automated rigid body registration and then align the MSI images to this. With SciLS, the sections are manually co-registered together based on the shape of the sections.

### Multimodal 3D Imaging

3D-MSI also opens the possibility of correlating the volumetric molecular information with data from other (3D) imaging modalities [76]. A common example is the combination of 3D MALDI-MSI data with standard histology techniques. Therefore, Lotz and coworkers analyzed 162 consecutive sections of an oral squamous cell carcinoma with MALDI-MSI, conventional hematoxylin and eosin, and immunohistochemistry. The transfer of these two modalities (MSI and optical microscopy) into the same coordinate system allowed all 3D data to be viewed next to each other, which led to a better understanding of the functional heterogeneity within the tumor as spatial context and progression can readily be determined from the H&E (Fig. 5a) [70]. The visual integration of MSI data with data from *in vivo* imaging techniques also harbors great potential by combining the molecular and anatomical information from different spatial scales. In this context, Abdelmoula *et al*. developed an automatic co-registration between 3D-MSI and MRI that tackles the differences in spatial scales and coordinate systems, the lack of direct spatial-correspondences, or nonlinear tissue deformations (Fig. 5b) [71].

### Spatial Statistics in 3D-MSI

3D-MSI data puts new demands on data analysis strategies itself. It is already known in 2D-MSI that the spatial context of the single mass spectra is a factor that can be exploited for multivariate analyses or has to be accounted for when performing inferential statistical analyses. Spatial-aware segmentation, as reported by Alexandrov and Kobarg [77], would have to be extended to the clustering of voxels. Likewise, statistical approaches that account for the inherent spatial autocorrelation in MSI data would have to be extended to the third dimension [78]. Morphometric characteristics of MSI data has been shown by Picard de Muller *et al*. to carry biomedical relevant information, which would also have to be calculated on voxels instead of 2D pixels [79].

Recently, Abdelmoula *et al.* have translated and extended the nonlinear t-SNE dimensionality reduction to the interactive visual exploration of 3D-MSI datasets using a hierarchical version of t-SNE. They have demonstrated that this approach can rapidly identify regions of interest within large high-dimensionality 3D-MSI datasets [80].

### Other Data Analysis Challenges in 3D-MSI

Other, less-apparent data analysis challenges remain, such as the calculation of average spectra across a 3D volume when serial sections are not evenly spaced. This requires the development of a weighted average procedure that uses the information of the *z*-position of all sections that are taken into account. A practical challenge is the annotation of tissue regions, already a huge bottleneck in 2D-MSI studies, and even more so in 3D-MSI studies, as multiple sections need annotation. Semi-supervised learning approaches where partial annotations by a pathologist are combined with deep learning on the MSI data are proposed for the classification of the remaining tissue by Inglese *et al.* [19]. If the sections are consecutive and have already been aligned to form a 3D model, one could also consider interpolating the partial annotations throughout the volume. In that light, one could also foresee the translation of the already existing approaches from unbiased tissue stereology to the molecular 3D-MSI data, where stringent sampling methods, and geometrical and statistical principles are used to obtain accurate and precise three-dimensional information [14]. Ultimately, novel data analysis approaches need to be developed that (a) will enable the researcher to extract the added information provided by the third dimension from a 3D-MSI dataset and (b) does not increase the manual workload for the researcher and maintains it similar to the 2D scale.

## Conclusion

Mass spectrometry imaging is an added tool for the spatially resolved analysis of molecules in biological tissue specimens in many fields of biomedical research. 3D-MSI studies are gaining momentum as the benefit of the added information from the 3rd dimension is slowly beginning to outweigh the additional experimental and post-experimental efforts. While the throughput is not a limitation anymore nor by sample preparation [33] nor by instrumentation speed [31], data handling, processing, and analysis remain bottlenecks. Dedicated software solutions exist but still cannot alleviate the additional data analysis workload that results from 3D studies. This holds especially true for histological annotations, spectral and image alignments, co-registration for integration with other imaging modalities, experimental outlier detection, and smart 3D visualizations. This has been recognized by the field shown by the many recent publications. Once these barriers are alleviated, 3D-MSI will be routinely extended to multisample studies. This results in a rapid three-dimensional, fully spatially aware molecular biology method that will cause a paradigm shift in patient diagnostics.
